# Glucagon-like Peptide-1 Receptor Agonists Associated Gastrointestinal Adverse Events: A Cross-Sectional Analysis of the National Institutes of Health All of Us Cohort

**DOI:** 10.3390/ph17020199

**Published:** 2024-02-02

**Authors:** Wafa Ali Aldhaleei, Tadesse M. Abegaz, Akshaya Srikanth Bhagavathula

**Affiliations:** 1 Division of Gastroenterology and Hepatology, Mayo Clinic, Jacksonville, FL 32224, USA; aldhaleei.wafa@mayo.edu; 2 Economic, Social and Administrative Pharmacy (ESAP), College of Pharmacy and Pharmaceutical Sciences, Institute of Public Heath, Florida A&M University, Tallahassee, FL 32307, USA; tadesse1.abegaz@famu.edu; 3 Department of Public Health, College of Health and Human Services, North Dakota State University, Fargo, ND 58108, USA

**Keywords:** glucagon-like peptide-1 receptor agonists, hypoglycemic agents, cardiovascular drugs, type 2 diabetes mellitus, obesity, adverse events, safety, gastrointestinal, All of Us, United States

## Abstract

Background: Glucagon-like peptide-1 receptor agonists (GLP-1 RAs) are commonly used diabetes and obesity medications but have been associated with gastrointestinal (GI) adverse events. However, real-world evidence on comparative GI adverse reaction profiles is limited. Objectives: This study aimed to evaluate GI adverse events among GLP-1 RA users and compare semaglutide, dulaglutide, liraglutide, and exenatide safety regarding the GI adverse reaction profile. Methods: This retrospective cross-sectional analysis utilized real-world data on 10,328 adults with diabetes/obesity in the National Institutes of Health All of Us cohort. New GLP-1 RA users were identified, and GI adverse events were examined. Logistic regression determined factors associated with GI adverse events. Results: The mean age of the study population was 61.4 ± 12.6 years, 65.7% were female, 51.3% were White, and they had a high comorbidity burden. Abdominal pain (57.6%) was the most common GI adverse event, followed by constipation (30.4%), diarrhea (32.7%), nausea and vomiting (23.4%), GI bleeding (15.9%), gastroparesis (5.1%), and pancreatitis (3.4%). Dulaglutide and liraglutide had higher rates of abdominal pain, constipation, diarrhea, and nausea and vomiting than semaglutide and exenatide. Liraglutide and exenatide had the highest pancreatitis (4.0% and 3.8%, respectively). Compared to semaglutide, dulaglutide and liraglutide had higher odds of abdominal pain, and nausea and vomiting. They also had higher odds of gastroparesis than semaglutide. No significant differences existed in GI bleeding or pancreatitis risks between the GLP-1 RAs. Conclusions: In this real-world cohort, GI adverse events were common with GLP-1 RAs. Differences in GI safety profiles existed between agents, with exenatide appearing safer than other GLP-1 RAs, except for gastroparesis. These findings can inform GLP-1 RA selection considering GI risk factors. Further studies are needed to evaluate the causal relationship and GLP-1 RA safety with concomitant medication use.

## 1. Introduction

Glucagon-like peptide-1 receptor agonists (GLP-1 RAs) are an injectable class of medications that are well established for use in managing type 2 diabetes mellitus (T2DM). Due to their glucose-dependent mechanisms of insulin secretion augmentation and glucagon suppression, GLP-1 RAs are able to effectively improve glycemic control in T2DM patients without substantially increasing the risk of hypoglycemia [1]. They work by lowering plasma glucose through effects on insulin and glucagon secretion, as well as by slowing gastric emptying, leading to a reduction in plasma glucose levels [2]. The interaction of GLP-1 and its receptors is known to act through the cAMP-PKA pathway, promoting the synthesis of ATP and the release of insulin granules into the blood through exocytosis [1]. These mechanisms contribute to the glucose-lowering effects of GLP-1 RAs, making them valuable in the treatment of T2DM. Moreover, GLP-RAs can also contribute to weight reduction by reducing appetite and the feeling of hunger. This occurs through directly stimulating POMC/CART neurons and indirectly inhibiting neuropeptide Y (NPY) and agouti-related peptide (AgRP) neurons, which can lead to reduced energy intake [3].

GLP-1 RAs comprise several compounds with varying pharmacokinetic properties, duration of action, and clinical effectiveness [1,2]. GLP-1 RAs have also demonstrated effectiveness for weight management and maintenance of weight loss in the treatment of people with overweight and obesity [4,5,6]. They are also used as an adjunct for weight loss in people without diabetes [7]. Some GLP-1 RAs have also been approved and marketed specifically for weight loss based on evidence that they reduce food intake by stimulating insulin and inhibiting glucagon secretion postprandially [4]. Treatment guidelines recommend the use of GLP-1 RAs due to their positive impact on T2DM complication prevention, blood pressure and lipid level improvement, and amelioration of sleep apnea [1,8,9,10].

However, despite these advantages, GLP-1 RAs have been associated with several gastrointestinal (GI) adverse events, though reported differences between specific agents are conflicting [11,12,13]. GI adverse events may range from mild to serious, including abdominal pain, nausea, vomiting, constipation, diarrhea, delayed gastric emptying, and pancreatitis [11]. Recent research has linked the use of GLP-1 RAs for weight loss to an increased risk of pancreatitis, gastroparesis, and bowel obstruction [14]. Under-recognition and delayed management of GI adverse events can exacerbate pre-existing GI and renal conditions [15]. One study found higher reporting odds of GI adverse events with semaglutide versus liraglutide [12], while a systematic review of case reports showed higher rates with liraglutide and exenatide [13]. Another study by Liu et al. reported higher odds of GI adverse events with semaglutide followed by liraglutide and dulaglutide [11]. Most prior studies were based on randomized controlled trials or U.S. FDA passive surveillance data, which may not represent real-world practice. Examining the association between GLP-1 RAs and GI adverse events at the population level is important to establish generalizable evidence. Therefore, this study aimed to examine the association between GI adverse events and patients using GLP-1 RAs in a representative sample from the National Institutes of Health (NIH)’s All of Us research program cohort. Using real-world data on a diverse population can provide additional insights into the comparative safety profile of specific GLP-1 RAs regarding GI tolerability.

## 2. Results

### 2.1. Cohort Description

**Figure 1:**
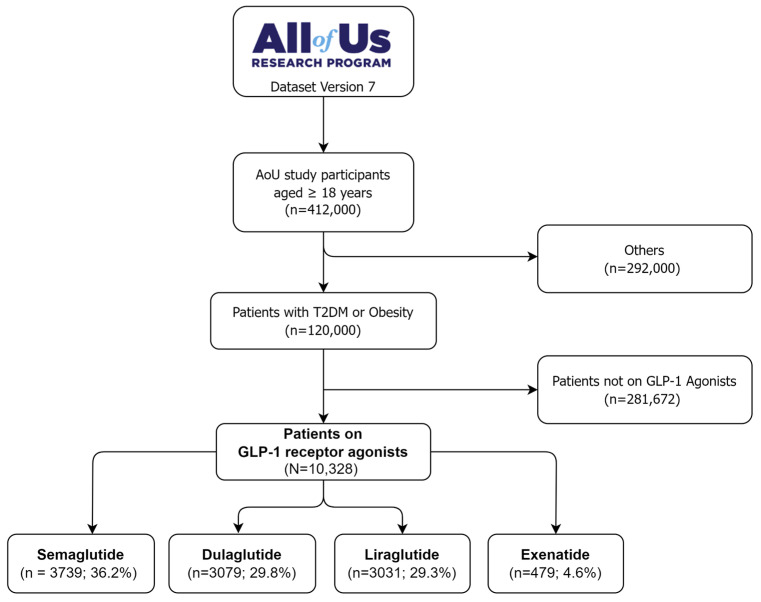
The flow diagram of selecting GLP-1 RAs users from the All of Us research database.

The study population was identified through a step-wise selection process from the All of Us research program database version 7, as depicted in Figure 1. The initial cohort comprised 412,000 patients aged 18 years or older. Only patients with a primary diagnosis of T2DM or obesity were considered (*n* = 120,000), while others were excluded (*n* = 292,000). The cohort was further restricted to those who received a GLP-1 RA prescription and used it for at least one month. This systematic identification process enabled the selection of a well-defined cohort of 10,328 new GLP-1 RA users with T2DM or obesity for analysis from the All of Us research program database. Of these, 36.2% were on semaglutide (*n* = 3739), 29.8% were on dulaglutide (*n* = 3079), 29.3% were on liraglutide (*n* = 3031), and 4.6% were on exenatide (*n* = 479).

### 2.2. Characteristics of the Study Population 

Table 1 presents the baseline demographic and clinical characteristics of the study cohort. The cohort included patients with T2DM (82.2%) or obesity (76.4%). The patients had a mean age of 61.4 ± 12.6 years and were predominantly female (65.7%) and White (51.3%), with a sizeable proportion of Black individuals (23.4%). Comorbidities included chronic kidney disease (CKD) (25.6%) and heart failure (HF) (19.2%). Mean lipase and amylase levels were 97.4 ± 2.9 U/L (normal level: 0–160 U/L) and 67.4 ± 5.6 U/L (normal level: 30–110 U/L), respectively.

### 2.3. GLP-1 RAs Associated GI Adverse Events

**Figure 2:**
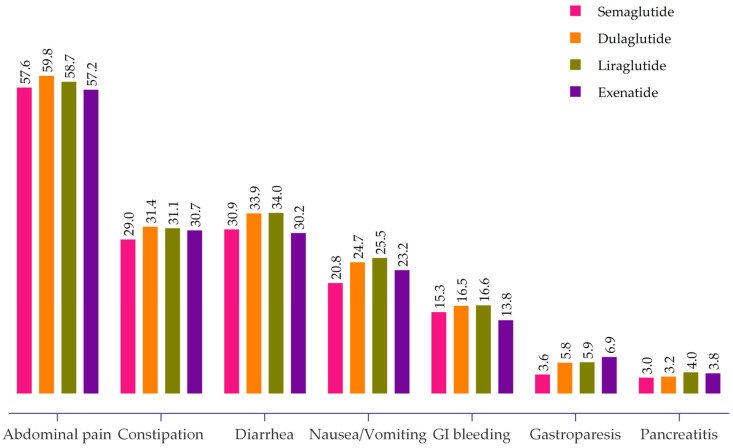
Proportion of GI adverse events associated with different GLP-1 RAs.

Overall, abdominal pain was the most common adverse event, reported in 57.6% (95% CI: 56.6–58.5%) of patients. Other prevalent GI adverse events included constipation (30.4%, 95% CI: 29.5–31.3%), diarrhea (32.7%, 95% CI: 29.5–31.3%), nausea and vomiting (23.4%, 95% CI: 22.6–24.2%), GI bleeding (15.9%, 95% CI: 15.2–16.6%), gastroparesis (5.1%, 95% CI: 4.6–5.5%), and pancreatitis (3.4%, 95% CI: 3.0–3.7%). The proportions of GI adverse events associated with different GLP-1 RAs in the study cohort are shown in Figure 2. Differences existed between the specific GLP-1 RAs. For instance, dulaglutide and liraglutide had higher rates of abdominal pain, constipation, diarrhea, and nausea and vomiting, while exenatide was associated with the highest gastroparesis prevalence (6.9%). Liraglutide and dulaglutide had the highest rates of GI bleeding (16.6% and 16.5%, respectively). Furthermore, liraglutide and exenatide had the highest rates of pancreatitis at 4.0% and 3.8% compared to dulaglutide (3.2%) and semaglutide (3.0%).

Table 2 outlines the prevalence of GI adverse events stratified by patient demographics and comorbidities among the study population. All GI adverse events were more common in women than in men and in Whites than in other races. GI adverse events increased in frequency with the presence of CKD and HF. For example, gastroparesis and pancreatitis were reported in 44.3% and 41.1% of those with CKD, respectively.

### 2.4. Factors Associated with GI Adverse Events among GLP-1 RAs Users

Factors associated with GI adverse events among GLP-1 RA users are presented and reported as adjusted odds ratio (aOR) with a 95% confidence interval (CI) in Table 3. Adjusted for sex, age, race, comorbidities, and medication type, male sex (aOR 0.50, 95% CI 0.46–0.55) and increasing age (aOR 0.98, 95% CI 0.97–0.99) were significantly associated with lower odds of abdominal pain. Similar findings were seen with diarrhea, nausea and vomiting, gastroparesis, and pancreatitis compared to women and younger patients. Compared to the Asian race, the White race had higher odds of diarrhea (aOR 1.71, 95% CI 1.23–2.54) and nausea and vomiting (aOR 2.11, 95% CI 1.33–3.51), while the Black race had increased odds of constipation (aOR 1.58, 95% CI 1.09–2.35). The presence of CKD and HF also increased the odds of all GI adverse events.

Compared to semaglutide, dulaglutide and liraglutide displayed higher odds of abdominal pain, and nausea and vomiting. Dulaglutide, liraglutide, and exenatide also had significantly increased odds of gastroparesis compared to semaglutide. No significant differences existed in GI bleeding risk or pancreatitis between the GLP-1 RAs.

## 3. Discussion

This real-world study of over 10,000 patients newly initiated on GLP-1 RAs aimed to evaluate some of the common and rare GI adverse events associated with these agents. We found high rates of abdominal pain, diarrhea, nausea, and other GI adverse events, especially among women and those with comorbidities. Differences existed between specific GLP-1 RAs regarding the risks of adverse events like gastroparesis and nausea and vomiting. Dulaglutide and liraglutide had higher GI adverse events than semaglutide and exenatide. These findings suggest variations in safety profiles between agents that should inform selection, particularly in patients with GI risk factors. Overall, our results from a large, diverse population align with prior evidence and provide further insights into the comparative GI tolerability of GLP-1 RAs in real-world practice [11,12,13,14,15].

Our findings showing higher GI adverse event rates with dulaglutide and liraglutide than semaglutide and exenatide are consistent with those of some prior studies. Liu et al.’s [11] analysis of FDA reports found higher severe GI events with liraglutide and dulaglutide, while Horowitz et al. [16] reported mostly mild side effects across agents, with more diarrhea in liraglutide users than exenatide. However, other studies conflict with our results. For example, Zhou et al.’s [12] pharmacovigilance study indicated that semaglutide had higher rates of multiple GI adverse drug reactions than liraglutide. Additional analyses found semaglutide was associated with the greatest risk for events like nausea and constipation compared to other GLP-1 RAs [11,12,14,17,18,19,20,21]. The reasons for discrepancies likely relate to differences in study design, data sources, and populations. Our real-world analysis of over 10,000 racially/ethnically diverse adults from the NIH All of Us research program, newly initiated on GLP-1 RAs, provides important complementary insights into comparative safety profiles, though findings remain mixed across studies. Further research is critical to clarify inconsistencies between observational studies, precisely identify predictors of GI events with GLP-1 RAs, and provide clarifying confirmatory or novel evidence about the safety profiles of these widely used T2DM medications. While challenging, achieving consistency across real-world analyses would strengthen confidence in the findings and better inform the clinical use of these agents.

Our study also identified that GI adverse event rates with GLP-1 RAs can vary based on several factors. Such factors included the specific drug used, patient demographics, and comorbidities. In this study, we found that compared to semaglutide, dulaglutide and liraglutide displayed higher odds of abdominal pain and nausea and vomiting. Dulaglutide, liraglutide, and exenatide also had significantly increased odds of gastroparesis compared to semaglutide. However, no significant differences were found in GI bleeding risk or pancreatitis between the GLP-1 RAs. In contrast, a real-world disproportionality study based on the FDA adverse event reporting system database found that semaglutide had the greatest risk of nausea, diarrhea, vomiting, constipation, and pancreatitis, while liraglutide had the greatest risk of upper abdominal pain [11]. The differences seen in the results across these studies may stem from variations in aspects such as the study design, the characteristics of the patient samples, and the analytical approaches used for data processing. For instance, the real-world study used data from the FDA adverse event reporting system, which includes reports from healthcare professionals, patients, and manufacturers, and may not be representative of the general population [11]. Furthermore, patient characteristics such as age, sex, race, and comorbidities can also influence the risk of GI adverse events. For example, male sex and increasing age were found to be significantly associated with lower odds of abdominal pain, diarrhea, nausea and vomiting, and pancreatitis compared to women and younger patients.

The high prevalence of GI events and differences in safety profiles between the GLP-1 RAs found in this real-world analysis should inform several aspects of clinical practice. The results highlight the need to consider patient risk factors like sex, age, race, and comorbidities when selecting a specific agent to mitigate adverse events. Clinicians should be aware that dulaglutide and liraglutide may confer greater GI adverse event risk compared than semaglutide or exenatide. Close monitoring for GI events is essential with any GLP-1 RA to ensure early recognition and appropriate management. Findings could eventually impact treatment guidelines or recommendations regarding first-line use or preferential prescribing in high-risk GI patients. Additionally, further investigation is needed through prospective studies confirming comparative safety, examining clinical outcomes related to GI events, and performing head-to-head comparisons of GLP-1 RAs.

Our study has several strengths and some limitations. To the best of our knowledge, this is the first population-based study in the United States (U.S.) evaluating the association between four GLP-1 RAs and GI adverse events. This is the largest and most diverse real-world study, comprising 10,328 people of an under-represented population. The study included participants with other comorbidities, such as CKD and HF, reflecting the general population to inform clinicians’ decision making and the appropriate selection of GLP-1 RAs given the GI risk profile. This is a cross-sectional study of an under-represented population that limits the establishment of causal inference and generalizability. Information obtained through electronic health records (EHRs) may inevitably result in implicit bias and misclassification. Additionally, GI adverse events related to concomitant medication use cannot be excluded. Therefore, future prospective studies with detailed medication profiles are warranted to inform causality, minimize study bias, and provide a comparative evaluation of GLP-1 RAs’ safety and clinical outcomes. Moreover, the All of Us research study participants were not selected based on the probability sampling method. Therefore, the findings of the current study should be interpreted with caution.

## 4. Materials and Methods

### 4.1. Study Design

This study was a retrospective cross-sectional analysis among All of Us participants aged ≥18 years at enrollment with EHRs information.

### 4.2. NIH All of Us Research Program

The All of Us research program was a national longitudinal cohort study led by the NIH that aimed to enroll over one million diverse participants across the U.S. [22,23,24]. The core goals were to combine electronic health records, surveys, biospecimen data, physical measurements, and digital health data from participants over time. By gathering extensive information on lifestyle, environment, genetics, and other factors influencing health, All of Us sought to enable individualized prediction, prevention, and treatment of disease. Key aspects included emphasizing the enrollment of minorities, returning research results to participants, and providing an open, collaborative data resource and tools to researchers. Through ensuring diversity and representation, All of Us strove to propel understanding of factors affecting health outcomes across populations. These initiatives have led to new insights on precision medicine and tackling health disparities [22,23,24,25]. The All of Us research program reviewed the enrollment demographics of existing cohorts and designated groups historically underrepresented, including those lacking healthcare access, with disabilities, and from rural areas. By including diverse populations, All of Us could conduct impactful research accounting for a breadth of demographic differences [26].

### 4.3. Study Population

We identified adult patients with newly diagnosed T2DM or obesity enrolled in the All of Us research program, one of the largest and most diverse biomedical cohorts within the U.S. The diagnoses in the All of Us research program are linked to the Observational Health and Medicines Outcomes Partnership common data model v5.3.1. The data came from a socioeconomically diverse cohort of over 400,000 American adults participating in the All of Us research program [22,25]. The All of Us research program institutional review board approved all study procedures. All participants provided written, informed consent to share EHRs with qualified investigators for broad-based research. The Controlled Tier v7 of All of Us database was accessed to collect the required data. This study followed the Strengthening the Reports of Observational Studies in Epidemiology reporting guideline.

### 4.4. Cohort Construction

Using prespecified eligibility criteria, two independent steps were established for this analysis. The first step involved identifying patients with incident T2DM or obesity who had no prior history of GI adverse events. The second step involved those who started GLP-1 RAs identified after the first prescription of these agents. The two steps were linked using concept sets, and the EHRs of each patient along with their sociodemographic characteristics were linked to create a final dataset in the researchers’ workspace. This dataset was opened using Jupyter notebooks. The final data were checked for completeness, and missing values on covariates were removed from the analysis.

### 4.5. Cohort Selection

#### 4.5.1. Inclusion Criteria

The inclusion criteria were:

- Aged 18 years or older.
- Incident T2DM or obesity as a primary diagnosis.
- Used any GLP-1 Ras for at least one month.
- Had complete medical data.

#### 4.5.2. Exclusion Criteria

The exclusion criteria were as follows:

- Type 1 diabetes mellitus.
- Gestational diabetes.
- Not on GLP-1 RAs therapy.
- Patients with missing data in their treatment records.
- Patients with the outcome of interest before treatment initiation.

### 4.6. Study Variables

For the purpose of this study, the following variables were accessed: sociodemographic details like sex, age, and race; clinical conditions—T2DM, obesity, CKD, and HF; laboratory parameters such as amylase and lipase; GLP-1 RA drugs—semaglutide, dulaglutide, liraglutide, and exenatide; and adverse GI events—abdominal pain, constipation, diarrhea, gastrointestinal bleeding, gastroparesis, nausea and vomiting, and pancreatitis. The pool of GI adverse events was adopted through a literature review [9,11,12,13,14,15] and U.S. FDA prescribing information [27]. To comply with the All of Us data and statistics dissemination policy, which aims to maintain confidentiality and protect participants’ privacy, variables with participant counts fewer than 20 were not distributed or published directly. Therefore, participants of Native Hawaiian, Middle Eastern, or more than one race were summed together and are displayed as one race group named “others”. Similarly, participants on lixisenatide were not included in the study analysis.

### 4.7. Statistical Analysis

All statistical analyses were performed using the cloud R program available in the researcher workspace in the All of Us research program database. The baseline characteristics of study participants are described using frequency counts and percentages for categorical variables, while means with standard deviations were used for continuous variables. The prevalence of gastrointestinal adverse events was compared between the cohorts using chi-squared tests. Multivariable logistic regression was used to determine factors associated with gastrointestinal adverse events, adjusting for sociodemographics, comorbidities, and medication type. We calculated the adjusted odds ratios (aORs) with 95% confidence intervals (CIs). A *p*-value < 0.05 was considered statistically significant for all analyses.

## 5. Conclusions

The findings of our study highlight that GLP-1 RA-related GI adverse events are common. GI safety profiles vary between agents, with exenatide appearing safer than other GLP-1 RAs, apart from gastroparesis. These results can guide clinicians in the decision making and the selection of GLP-1 RAs accounting for GI risk factors. However, these results should be interpreted with caution given this study’s limitations. Further studies are warranted to inform causality and provide a comparative evaluation of GLP-1 RA safety in relation to concomitant medication use.

## Figures and Tables

**Table 1:** The characteristics of the study population.

Characteristics	N = 10,328	Semaglutide (*n* = 3739)	Dulaglutide (*n* = 3079)	Liraglutide (*n* = 3031)	Exenatide (*n* = 479)
T2DM, %	8489 (82.2)	2904 (77.6)	2793 (90.7)	2343 (77.3)	30 (6.2)
Obesity, %	7886 (76.4)	2929 (78.3)	2183 (70.9)	2404 (79.3)	370 (77.2)
Sex, %					
Female	6785 (65.7)	2423 (64.8)	1937 (62.9)	2113 (69.7)	312 (65.1)
Male	3543 (34.3)	1316 (35.2)	1142 (37.1)	918 (30.3)	167 (34.9)
Age (Mean ± SD)	61.4 ± 12.6	61.7 ± 12.1	60.5 ± 12.3	60.9 ± 11.8	61.8 ± 11.9
Race, %					
White	5296 (51.3)	2058 (55.0)	1415 (45.9)	1590 (52.4)	233 (48.6)
Black	2414 (23.4)	846 (22.6)	790 (25.6)	662 (21.8)	116 (24.2)
Asian	185 (1.8)	80 (2.1)	54 (1.7)	47 (1.5)	4 (0.8)
Others	2433 (23.5)	755 (20.2)	820 (26.6)	732 (23.1)	126 (26.3)
Lipase levels (Mean ± SD)	97.4 ± 2.9	95.1 ± 3.0	100 ± 2.3	97 ± 2.5	94 ± 2.6
Amylase (Mean ± SD)	67.4 ± 5.6	68 ± 5.4	66 ± 5.3	66 ± 5.6	66.2 ± 5.1
Comorbidities, %					
CKD	2641 (25.6)	2947 (78.8)	2191 (71.1)	2222 (73.3)	327 (68.2)
HF	1982 (19.2)	620 (16.6)	609 (19.7)	643 (21.2)	119 (24.8)

T2DM: type 2 diabetes mellitus; CKD: chronic kidney disease; HF: heart failure. Other races included Native Hawaiian, Middle Eastern, or more than one race.

**Table 2:** GI adverse events based on patient characteristics.

	Abdominal Pain (*n* = 5949)	Constipation (*n* = 3144)	Diarrhea (*n* = 3374)	Nausea and Vomiting (*n* = 2421)	GI Bleeding (*n* = 1648)	Gastroparesis (*n* = 524)	Pancreatitis (*n* = 348)
Sex							
Female	4256 (71.5)	2274 (72.3)	2395 (71.0)	1857 (76.7)	1027 (62.3)	389 (74.2)	244 (70.1)
Male	1663 (27.9)	870 (27.6)	979 (29.0)	564 (23.3)	621 (37.7)	135 (25.7)	104 (29.8)
Race							
White	2810 (47.2)	1314 (41.8)	1761 (52.2)	1110 (45.8)	815 (49.5)	241 (46.0)	161 (46.2)
Black	1433 (24.1)	896 (28.5)	728 (21.6)	598 (24.7)	383 (23.2)	136 (25.9)	74 (21.2)
Asian	82 (1.3)	37 (1.2)	34 (1.0)	20 (0.8)	19 (1.1)	5 (0.9)	5 (1.4)
Others	1624 (27.3)	897 (28.5)	851 (25.2)	693 (24.6)	431 (26.1)	142 (27.1)	108 (31.0)
Comorbidities							
CKD	1737 (29.2)	1112 (35.3)	1153(34.2)	818 (33.7)	601 (36.4)	232 (44.3)	144 (41.4)
HF	1374 (23.1)	905 (28.8)	899 (26.6)	638 (26.3)	522 (31.6)	188 (35.8)	114 (32.7)

CKD: chronic kidney disease; HF: heart failure. Other races included Native Hawaiian, Middle Eastern, or more than one race.

**Table 3:** Factors associated with GI adverse events among GLP-1 RAs users.

	Adjusted Odds Ratio ^†^ (95% Confidence Interval)						
	Abdominal Pain	Constipation	Diarrhea	Nausea and Vomiting	GI Bleeding	Gastroparesis	Pancreatitis
T2DM	1.91	1.53	1.81	2.31	1.34	4.81	2.45
	[1.7–2.1] **	[1.32–1.78] *	[1.61–1.91] *	[1.82–2.41] **	[1.13–1.60] **	[3.41–7.82] **	[1.59–3.98] **
Obesity	2.50	1.97	2.11	2.04	2.50	1.31	1.45
	[2.1–2.6] **	[1.72–2.31] *	[1.59–2.29] **	[1.41–2.71] *	[1.98–2.74] **	[1.01–1.71] *	[1.05–2.03] *
**Sex**							
Female	1	1	1	1	1	1	1
Male	0.50	0.57	0.59	0.47	1.10	0.53	0.69
	[0.46–0.55] **	[0.51–0.67] **	[0.53–0.65] **	[0.40–0.51] **	[0.97–1.23] **	[0.43–0.66] **	[0.53–0.89] **
**Age**	0.98	1.00	0.99	0.98	1.00	0.97	0.99
	[0.97–0.99] **	[0.93–1.2]	[0.97–1.2]	[0.96–0.99] **	[0.97–1.1]	[0.96–0.99] **	[0.98–1.00]
**Race**							
Asian	1	1	1	1	1	1	1
White	1.15	1.01	1.71	2.11	1.17	1.61	1.82
	[0.84–1.45]	[0.73–1.49]	[1.23–2.54] **	[1.33–3.51] **	[0.73–1.97]	[0.71–4.51]	[0.65–7.38]
Black	1.20	1.58	0.96	1.89	1.14	1.41	1.40
	[0.86–1.63]	[1.09–2.35] **	[0.79–1.12]	[1.21–3.22] *	[0.71–1.97]	[0.62–4.21]	[0.49–5.75]
Others	1.75	1.64	1.80	2.48	1.34	0.69	2.38
	[1.27–2.41] **	[1.11–2.43] **	[1.31–2.81] **	[1.51–4.52] *	[0.83–2.21]	[0.23–4.52]	[0.85–9.69]
**Comorbidities**							
CKD	1.44	1.63	1.71	1.78	1.38	1.99	1.59
	[1.3–1.6] **	[1.46–1.83] **	[1.54–1.96] **	[1.54–2.41] **	[1.22–1.57] **	[1.63–4.23] **	[1.22–2.07] **
HF	1.55	1.74	1.51	1.54	1.73	1.90	1.63
	[1.4–1.8] **	[1.4–1.91] *	[1.34–1.93] **	[1.36–1.78] **	[1.5–1.97] **	[1.5–2.35] **	[1.24–2.14] **
**Drugs**							
Semaglutide	1	1	1	1	1	1	1
Dulaglutide	1.19	1.10	0.97	1.17	0.91	1.43	0.91
	[1.05–1.23] *	[0.99–1.96]	[0.71–1.95]	[1.03–1.34] *	[0.90–1.25]	[1.21–1.95] *	[0.68–1.23]
Liraglutide	1.12	0.99	0.89	1.19	1.03	1.51	1.21
	[1.01–1.24] *	[0.85–1.23]	[0.79–1.12]	[1.01–1.45] *	[0.92–1.2]	[1.21–1.91] *	[0.92–1.61]
Exenatide	0.75	0.88	0.91	1.10	1.04	1.65	1.04
	[0.59–1.73]	[0.71–1.89]	[0.79–1.73]	[0.79–2.14]	[0.57–1.73]	[1.10–2.41] *	[0.59–1.73]

* *p* < 0.05; ** *p* < 0.01; ^†^ adjusted for sex, age, race, medication type, comorbidities such as T2DM, CKD, HF, and obesity. Other races included Native Hawaiian, Middle Eastern, or more than one race.

## Data Availability

Data are available through the All of Us database.
